# Prostatic Leiomyoma: A Case Report

**DOI:** 10.1155/2012/702762

**Published:** 2012-11-11

**Authors:** Soufiane Mellas, Ahmed Amine Bouchikhi, Mohammed-Fadl Tazi, Abdelhak Khallouk, Jalal-Eddin Elammari, Mohammed-Jamal El Fassi, Moulay Hassan Farih

**Affiliations:** ^1^Department of Urology, Hassan II University Hospital of Fez, Fez 30020, Morocco; ^2^Department of Anatomy, Faculty of Medicine of Fez, Fez 30020, Morocco

## Abstract

Prostatic enlargement due to benign adenomatous hyperplasia is very common in elderly males. However, benign mesenchymal tumors especially true leiomyoma of the prostate are rare. We describe a 68-year-old male presenting a urinary obstruction lasting more than two years. The patient was referred for an acute urinary retention. The clinical examination was normal. The perrectal examination revealed an enlarged prostate without abnormalities. An endoscopic resection was performed. The histopathological examination revealed a benign smooth muscle tumor with absence of glandular hyperplasia; the result was confirmed by immunohistochemistry. Accordingly, the diagnosis of true leiomyoma of the prostate was made.

## 1. Introduction

Leiomyoma is a benign tumor arising from smooth muscle fibers. This tumor affects the prostate gland in two forms. The first form is the most frequent; it presents as small nodules leiomiomatosis associated with benign hyperplasia. The second form is the pure leiomyoma of the prostate; this form is avery rare entity [1]. True prostatic leiomyoma has been defined by Kaufman and Berneike as a smooth muscle tumor within the prostate or juxta-prostatic position, devoid of glandular elements [2]. The recognition of leiomyoma is important because of the potential of malignancy in such cases, and histopathology is the only tool to do so [1]. The goal of our paper is to report a prostatic leiomyoma with avery good outcome.

## 2. Case Report

A 68-year-old male was referred to our department while presenting acute urinary retention. A urethral catheter was set up. The pathological background of the patient revealed diabetes mellitus type 2 and high blood pressure and urinary obstruction lasting more than two years.

The per-rectal examination revealed an enlarged prostate without abnormalities. The physical examination was insignificant. The blood assessment was normal. PSA was at 0.3 ng/mL. The urine culture showed an infection with *E. coli*. A complete endoscopic resection was performed without complications.

**Figure 1:**
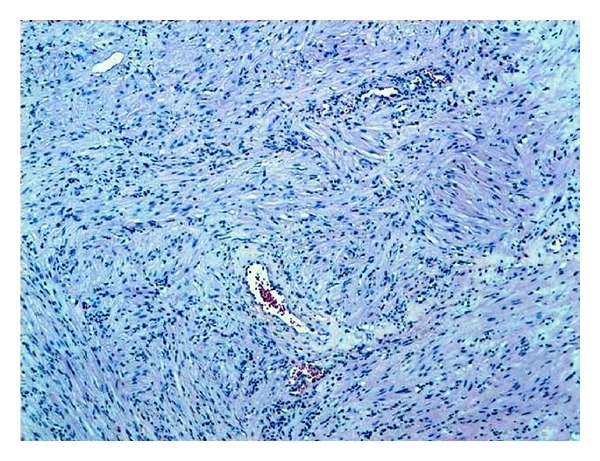
Benign tumoral proliferation organized in fascicles.

**Figure 2:**
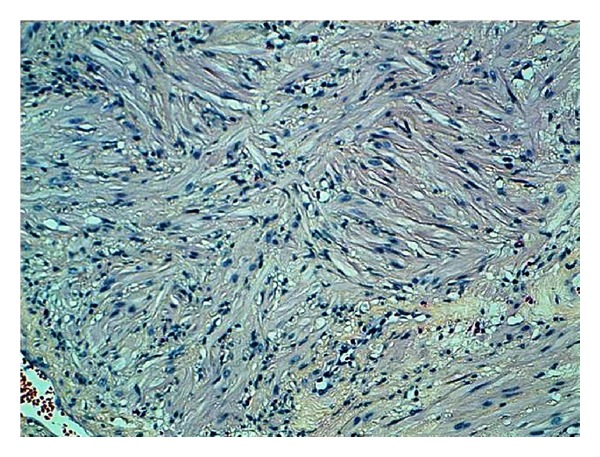
Spindle cells of smooth muscle with abundant cytoplasm and without nuclear atypia or mitosis (HES ×20).

The pathology report revealed the existence of pure leiomyoma of prostate. The microscopy showed the proliferation of spindle cells, without nuclear atypia or mitosis (Figure 1). While no epithelial tissue was demonstrated (Figure 2). The immunohistochemical techniques with desmin showed an intense intracytoplasmic positivity.

## 3. Discussion

Leiomyoma develop in all organs containing smooth muscles. It is more common in the gastrointestinal tract and the female genital tract [1].

Leiomyoma appears simultaneously in several localizations of human body, such as in the genitourinary system; it has been described in the kidney, ureter, bladder, urachus, prostate, urethra, and seminal vesicles [3, 4]. The first case of pure prostatic leiomyoma was described by Lebec et al., in 1876 [5]. Afterwards, 64 cases have been reported in the literature [6].

The pathogenesis is still unknown. Authors support that prostatic leiomyoma is exclusively originating from embryonic Müllerian remnants [1], while leiomiomatosis nodules associated with benign hyperplasia are the result of chronic inflammatory and infectious processes that cause the replacement of glandular tissue by smooth muscle [4].

The clinical presentation prostatic leiomyoma is identical to the benign prostatic hyperplasia. The finale diagnosis is based on histological arguments. While there is no specific clinical, biological, and imaging characteristic's. The pathological diagnosis criteria of pure prostatic leiomyoma are abscence of glandular component, presence of capsule, or pseudocapsule weighting more than 1 gram [1, 3].

The prognosis of these tumors is excellent because of its benign nature and lack recurrence after the complete removal [1, 7].

## 4. Conclusion

True leiomyoma is a rare tumor of the prostate that is diagnosed only by the histopathological examination. This pathological entity does not have any clinical, biological, or imaging characteristics. The treatment consists of a complete surgical excision. The prognosis, after appropriate treatment, is excellent without recurrence.
